# Grey matter volume in early human visual cortex predicts proneness to the sound-induced flash illusion

**DOI:** 10.1098/rspb.2012.2132

**Published:** 2012-10-24

**Authors:** Benjamin de Haas, Ryota Kanai, Lauri Jalkanen, Geraint Rees

**Affiliations:** 1 University College London Institute of Cognitive Neuroscience, 17 Queen Square, London WC1N 3BG, UK; 2 Wellcome Trust Centre for Neuroimaging, University College London, 12 Queen Square, London WC1N 3AR, UK

**Keywords:** multisensory, visual cortex, individual differences, voxel-based morphometry, sound-induced flash illusion

## Abstract

Visual perception can be modulated by sounds. A drastic example of this is the sound-induced flash illusion: when a single flash is accompanied by two bleeps, it is sometimes perceived in an illusory fashion as two consecutive flashes. However, there are strong individual differences in proneness to this illusion. Some participants experience the illusion on almost every trial, whereas others almost never do. We investigated whether such individual differences in proneness to the sound-induced flash illusion were reflected in structural differences in brain regions whose activity is modulated by the illusion. We found that individual differences in proneness to the illusion were strongly and significantly correlated with local grey matter volume in early retinotopic visual cortex. Participants with smaller early visual cortices were more prone to the illusion. We propose that strength of auditory influences on visual perception is determined by individual differences in recurrent connections, cross-modal attention and/or optimal weighting of sensory channels.

## 1. Introduction

When a single flash is accompanied by a rapid series of two or more bleeps, a perceptual ‘fission’ of the flash sometimes occurs and it is incorrectly perceived as multiple flashes [1]. The illusion is a striking example of how sounds can modulate visual perception (and thus challenges older theories of visual dominance [2]). It is in line with recent theories that propose statistically optimal weighting of sensory channels [3,4]. According to these theories, the weighting of sensory channels that together form an integrated percept depends on their relative levels of signal-to-noise ratio. In case of the sound-induced flash illusion, the number of events is easier to tell for auditory beeps than for visual flashes [4]. This also fits with other findings, showing that hearing has greater temporal resolution than vision (e.g. [5]; while vision has greater spatial resolution than hearing [6]).

However, perception of multisensory stimuli varies not only with stimulus properties, but also varies across observers. The same stimulus can evoke cross-modal effects reliably in some participants, but not in others. This can be seen for the sound-induced flash illusion [7], as well as for the McGurk illusion [8]. Individual differences in proneness to the McGurk illusion are correlated with the amplitude of blood-oxygen-level-dependent (BOLD)-signal responses to cross-modal stimuli in the left superior temporal sulcus (STS) [9,10]. Moreover, individual proneness to the sound-induced flash illusion is correlated with the degree to which sounds modulate visual event-related responses [7]. However, the neural basis of this variance in proneness to audio-visual interactions is still unclear.

Here, we tested whether individual proneness to an audiovisual illusion was correlated with differences in brain structure. Variability in several aspects of visual perception is correlated with differences in local brain structure (for a recent overview, see [11]). For instance, individual differences in the surface area of early visual cortex are correlated with individual differences in proneness to illusory size perception [12]. However, individual differences in the degree of cross-modal interactions have not previously been linked with variability in brain structure. Here, we tested whether proneness to the sound-induced flash illusion was correlated with differences in regional grey matter (GM) volume as measured using voxel-based morphometry (VBM; [13]). Experience of the ‘sound-induced flash illusion’ is accompanied by enhanced activity in retinotopically defined primary visual cortex (V1), superior colliculus (SC) and STS [14,15]. Furthermore, electroencephalography source localization [7] and short latencies of event-related magnetic field responses [15] suggest a role of auditory cortex in the illusion. Therefore, we hypothesized that individual differences in susceptibility to the sound-induced flash illusion would be reflected in structural variation of these regions across individuals.

## 2. Methods

### (a) Participants

A total of 29 subjects from the University College London (UCL) participant pool (20 females, aged 18 to 42 years; mean: 25 years, s.d.: 6 years) took part in this study. All participants completed the behavioural study outside the scanner and underwent the anatomical magnetic resonance imaging (MRI) scan on a different day.

### (b) Stimuli

**Figure 1.**
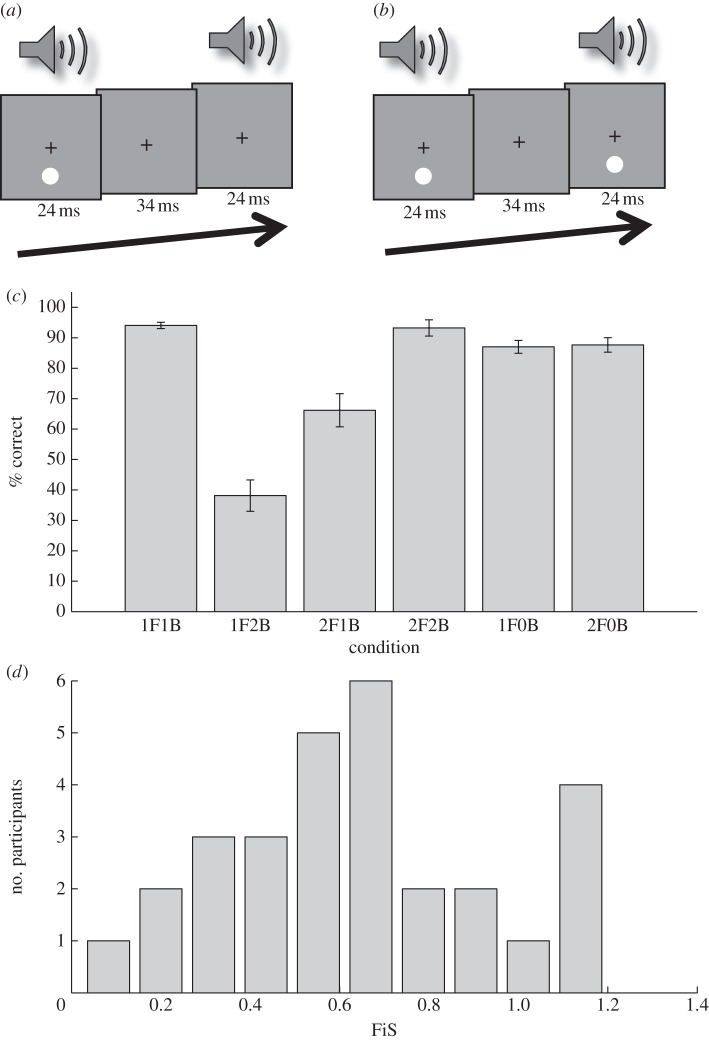
(*a*,*b*) Stimulus sequences and (*c*,*d*) behavioural results. The visual stimulus consisted of a visual disk (with a radius of 1° visual angle) that flashed once or twice at 5° eccentricity below or above the fixation cross, which was placed at the middle of the screen. The flash or flashes were combined with zero, one, or two beeps (3.5 KHz at 65 dBA). (*a*)  The critical trial type for the sound-induced flash illusion: one visual flash is accompanied by two beeps (1F2B). (*b*) A trial in which two flashes were accompanied by two beeps (2F2B). (*c*) Behavioural results by trial type. In each trial, participants (*n* = 29) indicated whether they saw one or two flashes. Bars represent the percentage of correct answers by trial type, averaged across participants. xFxB refers to number of flashes and beeps, respectively (from left to right: trials with one flash and one beep; one flash and two beeps; two flashes and one beep; two flashes and two beeps; one flash only; two flashes only). Error bars represent the s.e. of the mean. Note that the second bar represents the critical trial type (one flash, two beeps). The low overall proportion of correct answers for this trial type (38%) indicates that participants perceived the illusory second flash in the remaining trials of this condition (cf. table 2). (*d*) The distribution of FiS across the sample.

The visual stimulus consisted of a uniform white disk (140 cd m^−2^) that flashed for 24 ms (two frames at 85 Hz) on a uniform grey background (90 cd m^−2^) on a cathode ray tube monitor. The disk diameter was 2° visual angle, and it was placed at 5° eccentricity directly above or below a fixation cross that was displayed at the middle of the screen. The auditory stimulus consisted of a pure tone at 3.5 kHz that was played for 20 ms at 65 dBA on speakers adjacent to the monitor. All stimuli were programmed and presented in Matlab (Mathworks, Ltd) using the Cogent Graphics (http://www.vislab.ucl.ac.uk/cogent.php) and Psychophysics Toolbox 3 extensions ([16,17], http://psychtoolbox.org). In each trial, either one, or two flashes were presented, accompanied by either no, one, or two beeps, resulting in six trial types (1F0B, 2F0B, 1F1B, 2F1B, 1F2B and 2F2B were xFxB stands for the number of flashes and beeps, respectively). The onsets of flashes and beeps were synchronous. In trials with a second flash and/or second beep, the onset of the second event was time-locked to 34 ms after the offset of the first flash (see figure 1*a,b*).

### (c) Procedure

Participants sat on a chair in front of the monitor at 65 cm distance. They were asked to indicate whether they saw one or two flashes after each trial pressing either ‘1’ or ‘2’ on a numerical keypad with the index and middle finger of their right hand (in a time-window lasting 1800 ms after the stimulus presentation). Participants were advised they could ignore the beeps. Trials were presented in blocks of 102 trials with counterbalanced number of trial types in random order per block. A block lasted about five minutes, and participants were encouraged to take breaks in between blocks. The position of the visual stimulus (above or below fixation) was consistent within each block and changed (counterbalanced) between blocks. Each participant completed four to six blocks.

To ensure that participants kept fixation throughout a block their eye movements were monitored with an eyetracker system (Cambridge Research Systems). For 21 participants, eye movement data were fed into the stimulus presentation script online. For the programme to present the next trial, participants had to keep fixation for at least 500 ms. Fixations had to be within a square region of 3 × 3 around the fixation cross. For the remaining eight subjects, eye-data were analysed offline. For those participants, trials were excluded from analysis if the eyetracker did not record eye-position. Of the remaining trials (85.13%, s.d.: 13.54%), we included trials if participants' fixation did not deviate more than 1.5° from the midpoint of the screen on the vertical axis (95.41%, s.d.: 5.01%).

### (d) Analysis of behavioural data

All statistical analyses of the behavioural data were performed in Matlab (Mathworks, Ltd) and Pasw v. 18.0 (SPSS inc./IBM). To test for the sound-induced flash illusion, we compared the proportion of correct answers between conditions with a repeated measures ANOVA and post hoc *t*-tests. To determine proneness to the illusion, a ‘fission score’ (FiS) was calculated for each subject. It was defined as one minus the proportion of correct answers in the illusion trials (pCorr(1F2B)) and corrected for any response bias to report two flashes independent of the number of beeps:
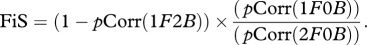


Additionally, the FiS was calculated separately for the two stimulus positions (above or below the fixation cross). The mean, range and variance for FiS and the simple proportion of correct trials were determined. Furthermore, the correlation between FiS for trials in which the disk flashed above and below the fixation cross, respectively, was calculated.

### (e) MRI data acquisition and pre-processing

T1 anatomical images of the brain were obtained with a 1.5 T Siemens Sonata MRI scanner (Siemens Medical). High-resolution anatomical images were acquired using a T1-weighted, three-dimensional, modified, driven-equilibrium Fourier transform sequence (repetition time = 12.24 ms; echo time = 3.56 ms; field of view = 256 × 256 mm; voxel size = 1 × 1 × 1 mm).

T1-weighted magnetic resonance images were first segmented for GM and white matter (WM) using the ‘New Segment’ segmentation tools in Statistical Parametric Mapping 8 (SPM8, http://www.fil.ion.ucl.ac.uk/spm). Subsequently, we performed diffeomorphic anatomical registration through exponentiated lie algebra (DARTEL) in SPM8 for intersubject registration of the GM and WM images [18]. To ensure that regional GM volume was maintained after the registration, the registered images were modulated by the Jacobian determinant of the flow fields computed by DARTEL. The registered images were smoothed with a Gaussian kernel of 8 mm full-width at half-maximum and transformed to Montreal Neurological Institute (MNI) stereotactic space using affine and nonlinear spatial normalization implemented in SPM8.

### (f) Voxel-based morphometry: statistical analysis

To test for correlations between GM volume and illusion strength, multiple regression analyses were performed on the smoothed GM images. FiSs were entered as vectors of interest into the design matrix, while total GM volume, age and sex were included as regressors of no interest in the model to control for any differences in these variables.

To incorporate our *a priori* hypotheses concerning the brain structures that we predicted to be involved, region of interest (ROI) masks were created using the SPM anatomy toolbox (http://www.fz-juelich.de/inm/inm-1/spm_anatomy_toolbox) and MarsBaR (http://marsbar.sourceforge.net/). The first three ROIs were derived from the illusion-specific significant activations reported in [14]. This study reported significant activation of retinotopically defined V1, and of right posterior STS and the SC. Because we did not have retinotopic data for our VBM subjects and the size of V1 has relatively large inter-individual variation [12,19], we used histological maximum probability maps to combine the BA17 and BA18 regions into one mask [20]. Right posterior STS and SC were incorporated via a 10 and 4 mm radius sphere, respectively, centred on the stereotactic coordinates of the peak voxels reported in [14]: [54,−54,30] and [2,−30,0]. Primary auditory cortex was added as a ROI following [7] and as defined by histological maximum probability maps [20].

The average GM volume within the ROIs was derived with MarsBaR and correlated with FiSs (controlling for total GM volume, age and sex). We used Bonferroni correction to adjust statistical thresholds for multiple ROIs tested. To avoid violations of the assumption of normally distributed data, a logit transformation was applied to the GM volumes [13]. Normality plots and Lilliefors tests [21] were then performed to test the assumption of normality for the distribution of the logit transformed average GM volumes within the ROIs and for FiSs, (the assumption of normality could not be refuted for any of them: FiS: *D* = 0.10, *p* = 0.68, n.s.; BA1718 GM: *D* = 0.11, *p* = 0.49, n.s.; *r* pSTS GM: *D* = 0.07, *p* = 0.99, n.s.; SC GM: *D* = 0.09, *p* = 0.87, n.s.; primary auditory cortex GM: *D* = 0.15, *p* = 0.10, n.s.). Outside the ROIs, an additional exploratory whole-brain analysis was carried out, using a threshold of *p* < 0.05, corrected for multiple comparisons using the familywise error (FWE) rate.

## 3. Results

### (a) Behavioural results

The sound-induced flash illusion was replicated [1,14]. Participants on average answered correctly on 38 per cent (s.d.: 28%) of 1F2B trials, indicating that they perceived the illusion on average in 62 per cent of trials. The inter-individual range was 2–100%. A repeated measures ANOVA indicated a significant difference between conditions (*F*_2.50_ = 45.17, *p* < 0.001, Greenhouse-Geisser corrected; figure 1*c*; table 1). The proportion of correct answers was significantly lower in the 1F2B condition, when compared with all other conditions and indicated by post hoc paired *t*-tests. Ninety-five per cent confidence intervals of the differences indicated that this was a strong and robust effect (table 2). Whether the disc appeared in the upper or lower visual field did not alter the strength of the sound-induced flash illusion (FiS below: 0.64 (s.d.: 0.29), FiS above: 0.62 (s.d.: 0.36); paired *t*-test: *t*_28_ = 0.64, *p* = 0.53, n.s.). The likelihood of reporting the illusion was highly correlated within individuals and across trials in which the disk flashed above versus below the fixation cross (*r* = 0.84, *p* < 10^−7^). The distribution of FiSs across participants is given in figure 1*d*.

**Table 1.** Descriptive statistics for behavioural data. (Cells contain information regarding the proportion of correct answers by trial type (xFxB refers to number of flashes and bleeps, respectively; FiS: fission score (see §2); below/above: position of flashing disk relative to fixation cross, see §2; s.d.: standard deviation).)

	min	max	range	mean	s.d.
1F1B	0.79	1.00	0.21	0.94	0.06
1F2B	0	0.98	0.98	0.38	0.28
2F1B	0.04	0.98	0.94	0.66	0.29
2F2B	0.32	1.00	0.68	0.93	0.14
1F0B	0.57	1.00	0.43	0.87	0.11
2F0B	0.49	1.00	0.51	0.88	0.13
FiS	0.02	1.20	1.17	0.63	0.30
FiS below	0	1.25	1.25	0.64	0.29
FiS above	0.04	1.39	1.35	0.62	0.36

**Table 2.** Group analysis of illusion effect. (*t*-statistics with corresponding standard deviation, *p* values and 95% confidence intervals (CIs) for paired *t*-tests contrasting the 1F2B condition with all other conditions. Note that the proportion of correct answers was significantly lower compared with all other conditions (cf. figure 1*c*). 95% CIs of the difference are given as [lower boundary, upper boundary].)

contrast	*t*_28_	s.d. (*t*)	95% CI	*p*
1F2B versus 1F1B	−11.24	0.27	[−0.66, −0.46]	<10^−11^
1F2B versus 2F1B	−4.86	0.31	[−0.40, −0.16]	<10^−4^
1F2B versus 2F2B	−9.11	0.33	[−0.67, −0.42]	<10^−9^
1F2B versus 1F0B	−10.01	0.26	[−0.59, −0.39]	<10^−10^
1F2B versus 2F0B	−10.07	0.26	[−0.60, −0.39]	<10^−10^

### (b) MRI results

**Figure 2.**
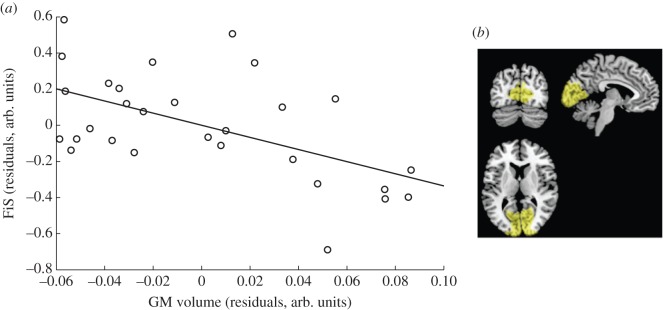
Correlation between proneness to the sound-induced flash illusion and GM volume (*a*) in early visual cortex (*b*). Each circle in (*a*) represents the BA17&18 GM volume and FiS of one participant (see §2 for details of FiS). The plot shows residuals after controlling for total GM volume, age and sex. Note that not controlling for age and sex, and using raw behavioural scores rather than FiS left the correlation qualitatively unchanged and statistically significant. The image to the right of (*b*) shows the corresponding BA17&18 ROI projected on slices from the coronal, sagittal and axial planes of a canonical T1 weighted structural image (‘collin27’, [22]). The image is in MNI stereotactic space. The mask was derived using histological maximum probability maps to combine the BA17 and BA18 regions into one mask (Eickhoff *et al.* [20]; see §2).

**Figure 3.**
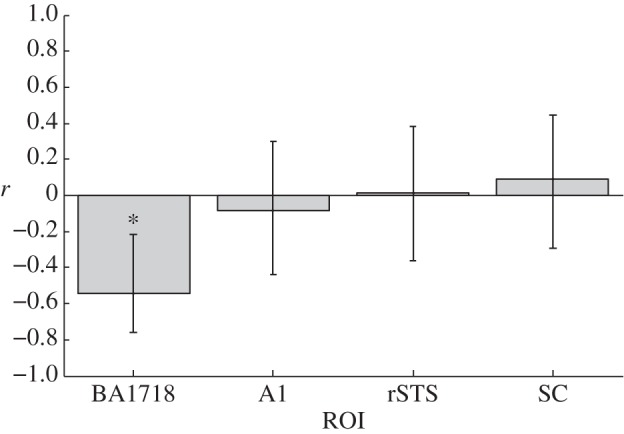
Correlations between proneness to the sound-induced flash illusion and GM volume in ROI (controlled for total GM volume, age and sex). Values on the *y*-axis reflect partial correlation coefficients. Error bars indicate 95% CIs of *r*, estimated using Fisher's *z*-transformation. The asterisk indicates *p* < 0.05 (Bonferroni corrected). ROI were BA17/18: early visual cortex (BA17 and 18 regions derived using Juelich Histological Atlas, see §2); A1, primary auditory cortex (derived using Juelich Histological Atlas, see §2); rSTS, right superior temporal sulcus (10 mm sphere centred on peak voxel reported by Watkins *et al.* [14]; see §2); SC, superior colliculus (4 mm sphere centred on peak voxel reported by Watkins *et al.* [14]; see §2).

The VBM analysis revealed a strong and statistically significant negative correlation between FiS and local GM volume in the BA17&18 ROI (controlled for global GM volume, age and sex): *r* = −0.55, *t*_24_ = −3.27, *p* = 0.01 (two-tailed and Bonferroni corrected for multiple ROIs; cf. figure 2). Note that this correlation remained qualitatively unchanged and statistically significant when not controlling for age and gender (*r* = −0.54, *t*_26_ =−3.30, *p* = 0.003) and when using raw behavioural scores (1-pCorr(1F2B)) instead of FiS (*r* = −0.47, *t*_26_ = −2.74, *p* = 0.01). No significant correlation between local GM volume and FiS was found for the primary auditory cortex, posterior STS and SC ROIs (cf. figure 3). Additional exploratory whole-brain analysis yielded no further significant findings at a threshold of *p* < 0.05, corrected for multiple comparisons (FWE correction).

To further test whether the correlation between proneness to the fission illusion and GM volume in the BA17&18 ROI was driven by the BA17 or BA18 region (or both), we correlated GM volume in each region separately with FiS (again controlled for global GM volume, age and sex). The GM volume of both regions was significantly negatively correlated with FiS; the relationship was slightly stronger for the BA18 mask (*r* = −0.60, *t*_24_ = −3.68, *p* = 0.001, two-tailed) than for the BA17 mask (*r* = −0.47, *t*_24_ = −2.64, *p* = 0.01, two-tailed), but this difference was not significant (*Z* = 0.97, *p* = 0.33, n.s.).

## 4. Discussion

We found reliable inter-individual differences in proneness to the sound-induced flash illusion. While some participants experienced the illusion hardly ever, others experienced it on almost every trial. Moreover, individual proneness to the illusion proved highly reliable across blocks with different flash locations, suggesting it to be a stable, trait-like feature.

We found a strong correlation between proneness to the illusion and local GM volume in early visual cortex (cf. figure 2). Participants with low GM volume in the BA17/18 region experienced the illusion significantly more often. Given we regressed out global GM volume, this points to a systematic relationship between individual proneness to the illusion and the relative amount of total GM dedicated to early visual cortex.

Higher proneness to the sound-induced flash illusion is associated with greater multisensory modulation of visual event related potentials (ERPs) [7]. In that earlier study proneness to the illusion correlated not only with the magnitude of illusion associated ERPs but also with supra-additive multisensory ERPs in trials with two beeps and two flashes (that did not induce any illusion). This result suggests increased proneness to the sound-induced flash illusion is associated with a more general enhancement of audiovisual integration. BOLD responses in V1 were found to be enhanced in an illusion specific way [14]. That is, BOLD responses in V1 were only enhanced by a second beep in trials in which it successfully induced the illusion. Taken together, these previous studies demonstrate that proneness to the sound-induced flash illusion correlates with stronger and more frequent multisensory modulations of early visual cortex activity. In light of these previous findings, our results predict that individuals with smaller visual cortices will exhibit stronger multisensory modulation of visual responses.

One possible explanation for greater multisensory effects being observed in anatomically smaller visual cortices may be incomplete scaling of multisensory connections with early visual cortex. Because visual cortex volume was controlled for total GM volume in our study, a smaller value implies a smaller proportion of GM dedicated to early visual processing. This might imply a greater ratio of multisensory GM (e.g. in STS)  versus visual GM and thus result in a greater number of multisensory synapses per visual neuron. This, in turn, would explain the higher likelihood for auditory modulation of visual perception, as indicated by proneness to the sound-induced flash illusion. However, this hypothesis should be taken with care and needs further testing. A post hoc test on our data could not confirm a significant correlation between the FiS, on the one hand, and the ratio of GM volume in the BA1718 mask  versus in the spherical right posterior STS mask, on the other (*r* = −0.18, *t*_25_ = −0.91, *p* = 0.37, n.s.).

An alternate hypothesis is that the weighting of sensory channels is tuned to the availability of neural resources. Our finding suggests that the relative amount of neural resources dedicated to the visual modality *in an individual brain* influences the weight placed on this sensory channel. Such a mechanism would be complementary to weighting mechanisms tuned to relative levels of input noise [3,23–25] as confirmed for the sound-induced flash illusion [4]. If the brain weights sensory channels according to their relative levels of *effective* noise, this will reflect more than input noise. It will also take into account the relative levels of *intrinsic* noise of sensory channels. One (well studied) aspect of such intrinsic noise is the general suitability of a sensory channel for the stimulus dimension at hand. This is reflected in general tendencies across participants, like visual dominance for spatial judgements [6] and auditory dominance for temporal judgements [5]. That is, the signal-to-noise ratio for spatial stimulus aspects is generally higher in the visual than in the auditory channel, whereas the opposite is true for temporal stimulus aspects.

Our results suggest an additional, more subtle aspect of intrinsic noise: it may vary between subjects according to the amount of GM dedicated to the specific sensory channel. Such a hypothesis would be in line with previous results, showing that the surface area of primary visual cortex in healthy humans is correlated with the cortical magnification factor at eccentricities comparable to that of our visual stimulus [26]. That is, subjects with smaller visual cortices have a visual representation that exhibits coarser spatial tuning. Further, this relationship between visual cortex area and acuity exists on the behavioural level as well. Cortical magnification within V1 is correlated with Vernier and grating acuity thresholds—across observers and eccentricities [27]. Taken together, previous results have shown that early visual cortex size correlates with the signal-to-noise ratio of visual representations. Our current finding converges with these results to suggest that the weighting of the visual channel in multisensory integration is tuned to the amount of GM dedicated to early visual cortex.

Variability in V1 surface area is negatively correlated with proneness to contextual visual-size illusions [12]. This result is interesting in the context of our study because it parallels the relationship between small visual cortex and high illusion proneness we found. This suggests that contextual influences are generally increased in small visual cortices—both, within and across modalities. Schwarzkopf *et al*. [12] interpreted their finding as pointing to a greater number of lateral connections from distant visual field representations within smaller visual cortices. One might speculate that our results might point to a similar neuroanatomical phenomenon, albeit across different areas of the brain rather than within one area. Increased contextual influence on visual processing might be owing to a higher degree of neural connectedness in small visual cortices—within and across areas as well as within and across modalities.

Finally, our results might be linked to individual differences in attention mechanisms [11,28]. Recent findings link the strength of the illusion to several such mechanisms. Specifically, top-down modality-specific attention shifts can suppress processing in the distractor modality and thereby attenuate the (visuo-tactile version of the) illusion [29]. Spatial attention directed away from the audiovisual stimuli diminishes early occipito-temporal components of the illusion-specific ERP components (which have been shown to be increased for participants experiencing the illusion more often [30]). Disruption of the angular gyrus with transcranial magnetic stimulation results in less frequent perception of the illusion, which has been attributed to attenuated effects of bottom-up attention evoked by the sounds [31]. Consequently, participants who are more prone to the sound-induced flash illusion could be more susceptible to auditory attentional capture, they could allocate more attention to the spatial position of the audiovisual stimuli or they could be less able to suppress the auditory modality via top-down attention. It is also interesting to speculate whether both the effects of cross-modal attention and the early visual cortex GM volume may be linked to effects of large network oscillatory phase reset [32,33].

Future experiments will shed more light on the mechanisms behind our finding. A putative relationship between early visual cortex size and its connectedness with multisensory areas can be tested using probabilistic tractography [34]. Functional definitions of early visual cortex (using retinotopic mapping, [35]) would allow dissociation of visual cortex area, and thickness and their respective association with proneness to the sound-induced flash illusion. To test the role of subtle differences in visual processing across participants, future studies could use sensitive visual tests, such as vernier acuity or a version of our purely visual trials, modified to enhance inter-individual variance in this condition (such as adding noise masks). A potential link between proneness to the illusion and differences in cross-modal attention could be tested behaviourally and followed up by tests on the individual propensity for cross-modal oscillatory phase reset [32,33].

In conclusion, we found a strong, negative correlation between early visual cortex GM volume and proneness to the sound-induced flash illusion. We proposed a neuroanatomical and functional explanations for this finding and ways to test these explanations in further experiments.
